# PRONAME: a user-friendly pipeline to process long-read nanopore metabarcoding data by generating high-quality consensus sequences

**DOI:** 10.3389/fbinf.2024.1483255

**Published:** 2024-12-20

**Authors:** Benjamin Dubois, Mathieu Delitte, Salomé Lengrand, Claude Bragard, Anne Legrève, Frédéric Debode

**Affiliations:** ^1^ Bioengineering Unit, Life Sciences Department, Walloon Agricultural Research Centre, Gembloux, Belgium; ^2^ Earth and Life Institute – Applied Microbiology, Plant Health, UCLouvain, Louvain-la-Neuve, Belgium

**Keywords:** long-read high-throughput sequencing, accuracy, clustering, polishing, duplex reads, microbiome, database, ribosomal operon

## Abstract

**Background:**

The study of sample taxonomic composition has evolved from direct observations and labor-intensive morphological studies to different DNA sequencing methodologies. Most of these studies leverage the metabarcoding approach, which involves the amplification of a small taxonomically-informative portion of the genome and its subsequent high-throughput sequencing. Recent advances in sequencing technology brought by Oxford Nanopore Technologies have revolutionized the field, enabling portability, affordable cost and long-read sequencing, therefore leading to a significant increase in taxonomic resolution. However, Nanopore sequencing data exhibit a particular profile, with a higher error rate compared with Illumina sequencing, and existing bioinformatics pipelines for the analysis of such data are scarce and often insufficient, requiring specialized tools to accurately process long-read sequences.

**Results:**

We present PRONAME (PROcessing NAnopore MEtabarcoding data), an open-source, user-friendly pipeline optimized for processing raw Nanopore sequencing data. PRONAME includes precompiled databases for complete 16S sequences (Silva138 and Greengenes2) and a newly developed and curated database dedicated to bacterial 16S-ITS-23S operon sequences. The user can also provide a custom database if desired, therefore enabling the analysis of metabarcoding data for any domain of life. The pipeline significantly improves sequence accuracy, implementing innovative error-correction strategies and taking advantage of the new sequencing chemistry to produce high-quality duplex reads. Evaluations using a mock community have shown that PRONAME delivers consensus sequences demonstrating at least 99.5% accuracy with standard settings (and up to 99.7%), making it a robust tool for genomic analysis of complex multi-species communities.

**Conclusion:**

PRONAME meets the challenges of long-read Nanopore data processing, offering greater accuracy and versatility than existing pipelines. By integrating Nanopore-specific quality filtering, clustering and error correction, PRONAME produces high-precision consensus sequences. This brings the accuracy of Nanopore sequencing close to that of Illumina sequencing, while taking advantage of the benefits of long-read technologies.

## 1 Introduction

The study of biological diversity has historically relied on direct observations and morphological studies to identify and classify organisms. This approach has not only proved to be labor-intensive but also limited in terms of accuracy and scope, particularly when dealing with highly similar species. The development of DNA sequencing transformed these methodologies, beginning with the first-generation sequencing techniques introduced with Sanger sequencing (Sanger and Coulson, 1975). This method allowed for reliable but low-throughput applications that could only process one isolated DNA sequence at a time. The DNA barcoding technique was then introduced, amplifying and sequencing a specific, short genetic marker within an organism’s DNA to facilitate species identification (Hebert et al., 2003).

Subsequently, second-generation sequencing technologies such as Roche 454 GS FLX and Illumina MiSeq and HiSeq systems brought significant advancements. These platforms enabled high-throughput sequencing (HTS), dramatically increasing data output and reducing costs. Despite their advantages, these technologies typically produced short reads, which presented challenges in terms of achieving high taxonomic resolution (Goodwin et al., 2016). Indeed, the amplicon length plays a crucial role in determining the accuracy and precision of taxonomic identification (Johnson et al., 2019; Nygaard et al., 2020). This limitation makes species differentiation more difficult, leading to variability in taxonomic classification and estimates of relative abundance based on the specific hypervariable region sequenced (Wasimuddin et al., 2020; López-Aladid et al., 2023).

The introduction of third-generation sequencing technologies marked a further evolution in the field. Platforms like Pacific BioSciences (PacBio) (Rhoads and Au, 2015) and Oxford Nanopore Technologies (ONT) (Deamer et al., 2016) offer long-read sequencing capabilities that overcome many of the limitations faced with earlier technologies (Malla et al., 2019). These long reads enhance taxonomic resolution and allow for more detailed genomic analyses, making them particularly valuable for studying complex multispecies communities (Jain et al., 2015). Full-16S sequencing showed good performances in increasing the taxonomic accuracy in complex multispecies communities (Jeong et al., 2021; Szoboszlay et al., 2023). Despite this huge technological innovation, it is not always possible to distinguish closely related species inside specific genera, such as *Clostridium* or *Pseudomonas* (Gürtler and Grando, 2013; Hu et al., 2022; Mulet et al., 2023). Going one step further, sequencing the entire 16S-ITS-23S region of the universally conserved rRNA operon (*rrn*) of bacteria and archaea allowed to achieve species (Cusco et al., 2018; Srinivas et al., 2024) or strain-level (Kerkhof et al., 2022) taxonomic resolution and capture evolutionary polymorphisms. Despite the higher raw read error rates initially associated with Nanopore technology, which posed a challenge for distinguishing closely related species (Jain et al., 2015), progress in chemistry, flowcell technology and duplex basecalling have substantially improved sequencing accuracy and quality (Deamer et al., 2016; Karst et al., 2021; Brown et al., 2023; Zhao et al., 2023).

Nevertheless, the challenges of analyzing such data underscore the inadequacies of existing bioinformatics pipelines, which are predominantly tailored for short-read sequences (Amarasinghe et al., 2020). Indeed, due to the unique characteristics of long reads, which include notably higher error rates and extended lengths, dedicated algorithms are essential for their correction (Zhang et al., 2020). Moreover, well-built and curated databases are of critical importance to capitalize on the taxonomic resolution brought by long read technologies. To mitigate these challenges and effectively harness the potential of Nanopore data, it is imperative to refine the data processing approaches for this new era of sequencing technology.

Our new bioinformatics tool, PRONAME (PROcessing NAnopore MEtabarcoding data), was designed to address these needs. This open-source, user-friendly pipeline optimizes the processing of raw Nanopore sequencing data and is adaptable to a range of biological taxa, from bacteria to fungi, plants and animals for instance, depending on the user’s focus. It includes two formatted full-16S databases and two *rrn* operon databases, providing robust support for diverse bacterial metabarcoding applications. Alternatively, the user can provide a custom database for any domain of life. Compared to existing pipelines, PRONAME offers enhanced accuracy and is the only one to take advantage of duplex reads associated with the new sequencing chemistry, demonstrating its efficacy and versatility in the genomic analysis of life’s diversity.

## 2 Materials and methods

### 2.1 The PRONAME pipeline

**FIGURE 1:**
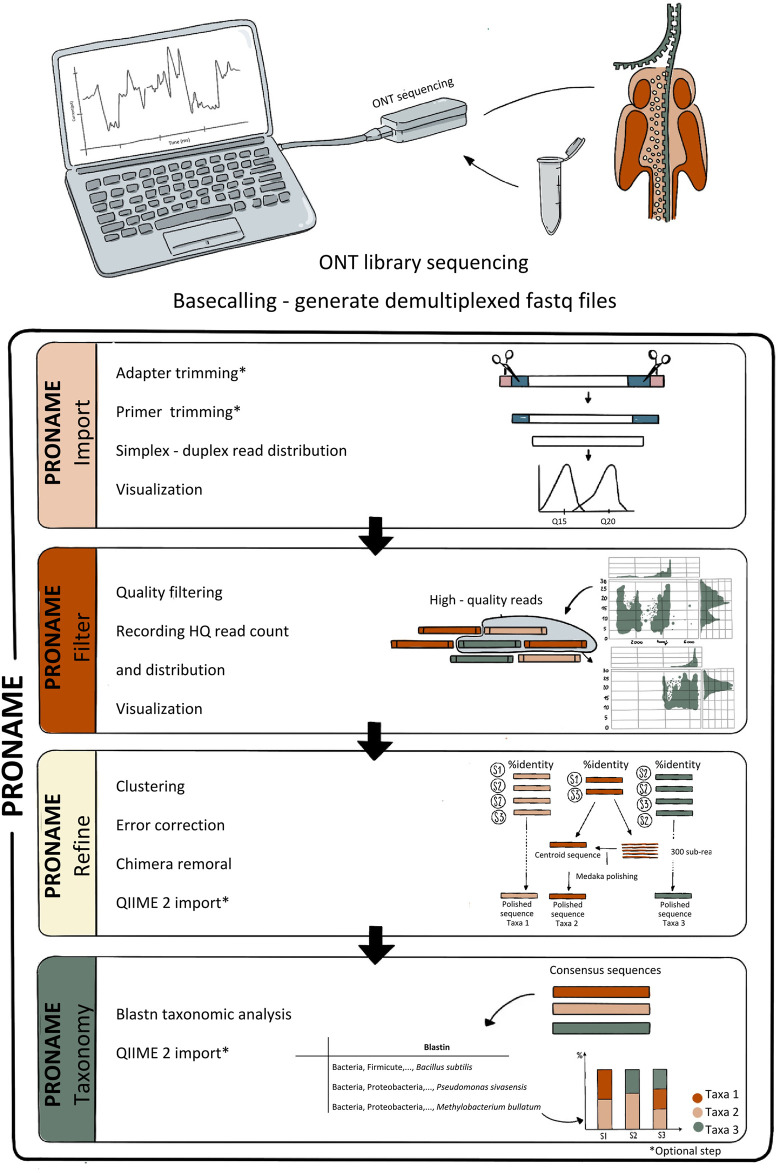
Schematic representation of the PRONAME pipeline. The pipeline comprises four scripts allowing importing and visualizing sequencing data (proname_import), quality-filtering data and keeping only duplex and/or simplex reads (proname_filter), significantly improving the accuracy by generating error-corrected consensus sequences and removing chimera (proname_refine), and performing the taxonomic analysis (proname_taxonomy). *Optional steps, the choice is left to the user whether or not to include them in the workflow.

The PRONAME pipeline has been written in Bash (GNU Project, 2024), with small companion scripts being written in Python (Python Software Foundation, 2024) and R (R Core Team, 2024). The whole pipeline and complementary files are available in our GitHub repository (https://github.com/benn888/PRONAME). For ease of use, PRONAME is provided as a Docker image (DockerInc., 2023). It simply needs to be pulled from Docker Hub to be directly useable, without installation and with all dependencies and databases available. Alternatively, the different pipeline scripts are also provided separately if the user wants to work outside the Docker environment. The PRONAME pipeline is made of four scripts as illustrated in Figure 1. The scripts must be run in the same order as they are presented below. As a first step, it is recommended that users access the help menu of each script or follow the GitHub tutorial to view the list of all arguments and usage examples.

#### 2.1.1 Proname_import

The first script of the PRONAME pipeline allows the import and initial processing of Nanopore metabarcoding data. The sequencing adapters are first trimmed off with Guppy (Guppy basecaller, v6.5.7) and primers are removed with Cutadapt (v4.9) (Martin, 2011). These two trimming steps are optional to offer greater flexibility regarding the type of input data. This script also includes the counting of simplex reads and duplex reads (if applicable) among each sample, as well as the creation of length-vs-quality scatter plots using NanoPlot (v1.43) (De Coster et al., 2018) for each type of reads (simplex, duplex and both). Scatter plot visualization is crucial for deciding which type of read to work with (duplex and/or simplex) and for defining the appropriate filtering thresholds to apply in the next script. However, as this can be a time-consuming step, an option has been added to skip this visualization step (although it is not recommended).

#### 2.1.2 Proname_filter

With this script, the user can take advantage of V14 sequencing chemistry introduced by ONT, which produces high-quality duplex reads exhibiting Q20+ quality (i.e., at least 99% average accuracy) and up to Q30 quality (99.9% average accuracy). This significant improvement in accuracy compared to simplex reads explains why the proname_filter script allows discarding simplex reads to work only with duplex data. Alternatively, it is also possible to execute the analysis with both simplex and duplex reads, or only with simplex reads (e.g., if a previous sequencing chemistry was used). NanoFilt (v2.8.0) (De Coster et al., 2018) is then used to filter reads using length and quality parameters provided by the user. The number of high-quality reads (i.e., simplex and/or duplex reads that passed the filtering step) is then reported and a new length-vs-quality scatter plot is built to evaluate the impact of the quality filtering process.

#### 2.1.3 Proname_refine

The proname_refine script generates high-accuracy consensus sequences in a multi-step process inspired by Ohta et al. (2023). High-quality reads are first clustered using VSEARCH (v2.22.1) (Rognes et al., 2016) according to a sequence similarity threshold provided by the user, and singletons are removed. Importantly, within each cluster, the read distribution and the link between each read and its sample provenance are recorded. This ensures that, at the end of the script, an OTU-like table reporting the frequency of every consensus sequence in each sample is generated. Seqkit (v2.3.0) (Shen et al., 2016) is used to extract from each cluster the centroid sequence on the one hand, and a subset of reads on the other hand. The error-correction is then performed with Medaka v2.0.1 (Medaka, 2024) by polishing each centroid sequence using its corresponding subset of reads. After a last filtering step to remove chimera sequences using VSEARCH, the user can choose whether to import the resulting representative sequences and corresponding frequency table into the QIIME2 platform (2024.5 release) (Bolyen et al., 2019).

#### 2.1.4 Proname_taxonomy

In this script, the user can employ one of the databases available with PRONAME (located in /opt/db/ in the Docker container) or provide a custom one, ideally formatted following the recommendations of Dubois et al., 2022. The taxonomic analysis is carried out outside QIIME2 with the blastn standalone tool (v2.15.0) (Camacho et al., 2009) from BLAST command line applications. This is due to the fact that the QIIME2 classify-consensus-blast plugin uses the blastn qcov_hsp_perc parameter and working on a per-HSP (high-scoring pair) basis does not seem to be the most relevant option when dealing with long-read data. Instead, the blastn results are filtered based on the blastn “qcov” specifier to keep only the matches showing an overall query cover percentage equal or higher than a threshold defined by the user (80% by default). This allows considering the query cover over the whole sequence (all HSPs), and hence provides results which are much more consistent with those from the online BLAST tool.

After analysis, the results can be reimported into QIIME2 to generate a taxa barplot. The user can take advantage of this to directly proceed to further processing within QIIME2, such as alpha-/beta-diversity analyses or differential abundance testing among others (see the tutorial in our GitHub repository). It is also possible to generate a phyloseq object (McMurdie and Holmes, 2013) composed of the consensus sequences, the OTU table, the associated taxonomy and a phylogenetic tree, in order to perform further analysis using R.

### 2.2 Reference databases

#### 2.2.1 Full-length 16S rDNA databases

The Silva 138 SSURef NR99 (Quast et al., 2012) full-length sequences and associated taxonomy were retrieved from the QIIME2 website (QIIME2 data resources, 2024) and exported into fasta and tsv files using the command ‘qiime tools export’. The sequence file was then formatted with the makeblastdb command to run with BLAST command line applications (BLAST® Command Line Applications User Manual, 2008). The Greengenes2 (McDonald et al., 2023) sequences and taxonomy were retrieved from their ftp site (Greengenes2 ftp, 2024). Given the large number of very small sequences in this database, sequences shorter than 900 bp were filtered out. Only sequences identified to the species level were kept. They were then formatted to match BLAST command line application requirements as detailed above.

#### 2.2.2 Ribosomal operon database

Some reference databases dedicated to the ribosomal RNA operon sequences do already exist, but they did not fit our purposes for several reasons, such as the region covered being too short, the number of sequences/represented species, their not up-to-date status, or how/whether they were curated. A new reference database was therefore developed and named the *rrn* operons Extracted from GENomes of Bacteria (rEGEN-B) database. This database in included in the Docker image and is also directly available at: https://doi.org/10.6084/m9.figshare.26380702. GenBank and RefSeq genomes were downloaded from the NCBI’s Genome resource page on 4 November 2024. The search was carried out to retrieve only assemblies with the “Chromosome” or “Complete” status, and with a year of release ranging from 2005 to 2024. Prokaryotic rRNA operon sequences were then extracted from these genomes using the sequences of 16S-27F (5′-AGRGTTYGATYHTGGCTCAG-3′) (Lane, 1991) and 23S-U2428R (5′-CCRAMCTGTCTCACGACG-3′) (Martijn et al., 2019) primers. The extraction was restricted to keep amplicons between 3,500 and 6,500 bp, allowing 10% mismatch. The script “extract_amplicons.sh” was written to carry out this *in silico* amplicon extraction; it is also available in our GitHub repository and in the Docker container (/opt/scripts/). The “data_summary.tsv” file, obtained at the same time as the download of the reference genomes, was used to retrieve genome taxids. The complete taxonomic lineages were recovered using the approach described in the DB4Q2 pipeline (Dubois et al., 2022). The database was then curated to filter out low-quality and suspected misidentified sequences, as described in the DB4Q2 pipeline. Briefly, reference sequences were analyzed in a cross-validation scheme with data leakage, i.e., where sets of test and training sequences are strictly identical. This enabled comparisons between expected and predicted taxonomies for each sequence and discarding those for which the expected taxonomy at the family rank was observed only once in the top 5 hits resulting from the blastn analysis. This database was complemented by the *rrn* operons extracted from the genomes of the 17 bacterial species from the mock community sequenced in this work (see below). The number of *rrn* copies per genome was recorded in Supplementary Material S1 to maintain a record. Duplicate copies of the *rrn* sequences from the same genome were then removed to reduce redundancy. The fasta file containing extracted operon sequences was then used to produce a database compatible with BLAST command line applications by applying the makeblastdb command. To enable users with lower computational capabilities to utilize the rEGEN-B database in a more efficient way, a lighter version of the database has also been compiled by extracting only the first copy of the *rrn* operon in each genome (see the “uniq” label in the database files). In complement to rEGEN-B, the recently published GROND database (Walsh et al., 2024) was also included in PRONAME as an optional database.

### 2.3 Mock community

#### 2.3.1 DNA extraction, library preparation and whole-genome sequencing

Seventeen Gram- and Gram + bacterial species were selected to assemble a mock community (Supplementary Material S2). Five of them belonged to the *Pseudomonas* genus and were deliberately chosen for their genetic proximity to put PRONAME to the test and evaluate its efficiency under very unfavorable conditions. DNA was extracted from pelleted bacterial cultures using the NucleoSpin Soil Kit (Macherey-Nagel), with SL1 and SX as lysis solutions. After purification with AMPure XP beads (Beckman Coulter), the DNA was visualized on 0.5% agarose gel and its concentration was measured using a Qubit 4 fluorometer (ThermoFisher). Sequencing libraries were then prepared using the Ligation Sequencing Kit V14 (SQK-LSK114) from Oxford Nanopore Technologies (ONT), and each library was loaded on a separate Flongle flowcell R10.4.1. The sequencing runs were performed using a MinION device and lasted 24 h.

#### 2.3.2 Genome assembly

The raw-signal POD5 reads were basecalled using Dorado (v0.3.4) (Dorado, 2023) with the dna_r10.4.1_e8.2_400bps_sup@v4.2.0 basecalling model. Adapters were removed using Guppy (v6.5.7) and genomes were then assembled using Trycycler (v0.5.4) (Wick et al., 2021). Briefly, short reads (less than 1,000 bp) and very bad reads (the worst 10%) were discarded. A third quality-filter was applied to remove reads with a Q score lower than 13. Reads were then sub-sampled into 12 read sets and the Flye (v2.9.2) (Kolmogorov et al., 2019), Miniasm (v0.3) (Li, 2016) + Minipolish (v0.1.2) (Wick and Holt, 2021) and Raven (v1.8.3) (Vaser and Šikić, 2021) assemblers were used to build four assemblies with each. The contigs of the 12 assemblies were clustered, reconciled, and a multiple sequence alignment was run on reconciled contig sequences. The sequencing reads were then partitioned between each cluster and a consensus contig sequence was generated for each cluster. Finally, Medaka (v1.8.0) was run on the Trycycler consensus sequences to polish them and further increase their accuracy.

The assembly metrics for the sequenced genomes of bacteria included in the mock community are reported in Table 1. The genome size ranged from 2.9 to 8.6 Mb, with a fluctuating number of plasmids (from 0 to 6). Notably, whereas every species displayed one circular chromosome, *Burkholderia anthina* happened to have three chromosomes, which allies with previous reports (Bochkareva et al., 2018). All sequenced genomes have been deposited on the NCBI under the BioProject numbers PRJNA1134685 and PRJNA1141912.

**TABLE 1:** Assembly metrics for the genomes of the 17 bacterial species sequenced in this work.

Species	Genome size (bp)^a^	Genome coverage	Number of *rrn* operon copies	NCBI BioSample accession number
*Glutamicibacter creatinolyticus*	3,377,278 (1C)	240x	5	SAMN42431379
*Staphylococcus equorum*	2,992,544 (1C + 6P)	135x	7	SAMN42431380
*Sphingomonas albertensis*	4,099,393 (1C)	82x	3	SAMN42431381
*Burkholderia anthina*	8,619,681 (3C + 1P)	81x	6	SAMN42431382
*Methylobacterium bullatum*	5,014,694 (1C + 1P)	65x	4	SAMN42431383
*Pedobacter foliorum*	6,131,055 (1C)	37x	6	SAMN42431384
*Enterobacter kobei*	4,814,064 (1C + 2P)	132x	8	SAMN42431385
*Sphingobacterium thalpophilum*	5,718,724 (1C)	111x	7	SAMN42431386
*Xanthomonas translucens*	4,658,813 (1C + 1P)	48x	2	SAMN42431387
*Microbacterium oxydans*	3,822,978 (1C)	168x	2	SAMN42431388
*Pseudomonas cichorii*	5,986,012 (1C)	194x	6	SAMN42431389
*Pseudomonas syringae*	5,947,987 (1C)	166x	5	SAMN42431390
*Pseudomonas asplenii*	6,658,430 (1C)	107x	6	SAMN42431391
*Pseudomonas sivasensis*	6,351,098 (1C)	197x	6	SAMN42230335
*Pseudomonas lurida*	6,039,873 (1C)	52x	5	SAMN42431392
*Bacillus subtilis*	4,228,867 (1C)	55x	10	SAMN42431393
*Pantoea agglomerans*	5,268,351 (1C + 4P)	57x	7	SAMN42431394

^a^ For each assembly, the number of chromosomes (C) and plasmids (P) is reported in brackets.

#### 2.3.3 Mock community constitution

The total genome length and the number of *rrn* operon copies were inferred from each assembled genome. These parameters and the concentration of DNA extracted from each bacterial culture were used to mix the DNA of the 17 members in the mock community with an identical number of *rrn* operon copies for each of them.

### 2.4 DNA metabarcoding assay

To illustrate the efficiency of the PRONAME pipeline and quantify the accuracy of consensus sequences it produces, six metabarcoding assays “RRN1” to “RRN6” were set up (Supplementary Material S3). The mock community DNA was amplified with four primer pairs covering the majority of the *rrn* operon (Table 2). Four assays were carried out with the current R10.4.1 flowcells (V14 chemistry), and two with the older R9.4.1 flowcells. The PCR reactions were performed in triplicate. All 25 µL-PCR reactions were carried out using 12.5 µL of 2X GoTaq® Long PCR Master Mix (Promega), 1.25 µL of 10 µM forward and reverse primers, 1 µL of DNA and 9 µL of nuclease-free water (QIAGEN). Triplicate PCR products were pooled during purification using AMPure XP Beads (Beckman Coulter). The amplicon quality was checked by running 5 µL of PCR products on 0.6% agarose gel and DNA concentration was measured using a Qubit 4 fluorometer (ThermoFisher). For the four assays run on R10.4.1 flowcells, the sequencing libraries were prepared using the Ligation Sequencing Kit V14 (SQK-LSK114). Each library was run for 24 h on a Flongle flowcell (R10.4.1) using a MinION device (Oxford Nanopore Technologies). Raw sequencing data were basecalled using Dorado (v0.3.4) with the dna_r10.4.1_e8.2_400bps_sup@v4.2.0 basecalling model. For the two assays using older flowcells and kit chemistry, the libraries were prepared using the Ligation Sequencing Kit (SQK-LSK110) and each was run for 24 h on a R9.4.1 Flongle flowcell. Raw sequencing data were basecalled using Dorado (v0.3.4) with the dna_r9.4.1_e8_sup@v3.6 basecalling model.

**TABLE 2:** PCR primers used in the six metabarcoding assays.

Metabarcoding assay name	Flowcell/Chemistry generation	Primer type	Primer name	Primer sequence (5′-3′)	Average amplicon length (bp)
RRN1	R10.4.1 flowcell, current chemistry (V14)	F	16S-27F	AGRGTTYGATYHTGGCTCAG	4,265
		R	23S-2241R	ACCRCCCCAGTHAAACT	
RRN2	R10.4.1 flowcell, current chemistry (V14)	F	16S-A519F	CAGCMGCCGCGGTAA	4,112
		R	23S-U2428R	CCRAMCTGTCTCACGACG	
RRN3	R10.4.1 flowcell, current chemistry (V14)	F	16S-27F	AGRGTTYGATYHTGGCTCAG	4,616
		R	23S-U2428R	CCRAMCTGTCTCACGACG	
RRN4	R10.4.1 flowcell, current chemistry (V14)	F	16S-8F	AGRGTTYGATYMTGGCTCAG	4,516
		R	23S-2490R	CGACATCGAGGTGCCAAAC	
RRN5	R9.4.1 flowcell, older chemistry	F	16S-27F	AGRGTTYGATYHTGGCTCAG	4,265
		R	23S-2241R	ACCRCCCCAGTHAAACT	
RRN6	R9.4.1 flowcell, older chemistry	F	16S-A519F	CAGCMGCCGCGGTAA	4,112
		R	23S-U2428R	CCRAMCTGTCTCACGACG	

The average amplicon length was computed by performing *in silico* PCR, with the extract_amplicons.sh script, using each of these primer sets and the 17 assembled genomes from the mock community as template.

## 3 Results

### 3.1 Developed reference databases

**FIGURE 2:**
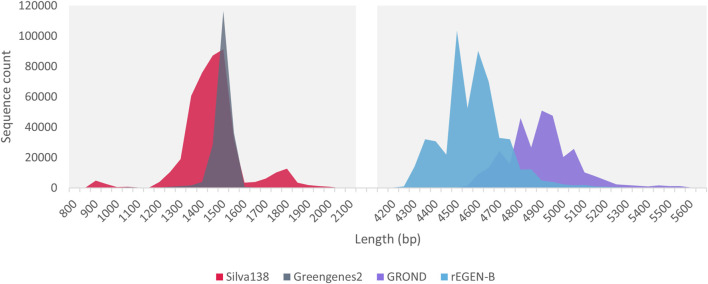
Sequence length distribution of the databases included in PRONAME. Silva138 and Greengenes2 are existing databases dedicated to the 16S rRNA gene that were formatted to fit within the PRONAME framework. rEGEN-B is a new curated database developed in this work and dedicated to bacterial *rrn* operon sequences (the 16S-ITS-23S region).

The sequence length distribution of the Silva138 and Greengenes databases developed/formatted in this work showed a clear peak around 1,500 bp (Figure 2). This corresponds to the length of the full 16S rRNA gene, which makes these databases useful for researchers performing full 16S metabarcoding with custom primers or directly with the ONT 16S Barcoding Kit. Regarding the databases dedicated to the *rrn* operon, the rEGEN-B and GROND sequences displayed much longer profiles, with distribution curves centered around 4,500 and 4,900 bp respectively. While GROND sequences correspond to the full *rrn* operon, rEGEN-B sequences do not include the last portion of the 23S gene. This region was considered less useful, since it is not amplified by common 16S/23S metabarcoding primers; therefore, it was excluded to save computation time. The wider shape of the length distribution peak compared to 16S databases is explained by the increased sequence length, offering more variation possibilities and in particular, the presence of the ITS region which known to display important length polymorphism.

As illustrated in Table 3, the Silva138 database displays more sequences and covers more species compared to Greengenes2, although its average sequence length is slightly shorter. Regarding the *rrn* operon reference dataset, the rEGEN-B and GROND databases demonstrated an increased number of sequences and represented species compared to other previously published databases. Interestingly, the average sequence length measured indicates that rEGEN-B reference sequences are long enough to cover the entire length of amplicons produced with the primer sets most commonly used in bacterial long-read metabarcoding analyses (Table 2). The rEGEN-B database is also the only one to match the “high-confidence curation” criteria that were defined in this work: (i) the sequences in the database only come from genomes with confident assembly levels (i.e., “chromosome” or “complete genome” status, but not “contig” nor “scaffold”), (ii) only sufficiently recent genomes were retained for operon sequence extraction (nothing before 2005), and (iii) the database was curated using the DB4Q2 pipeline to discard low-quality and misidentified sequences. The species-level accuracy of both *rrn* operon databases was also investigated with a cross-validation (CV) analysis using the QIIME2 command ‘qiime rescript evaluate-cross-validate’ (k-fold cross validation in a pseudo-realistic situation). For the GROND databases, we selected the one built with RefSeq sequences in order to compare databases with sequences from the same origin (since RefSeq was also used for rEGEN-B). The rEGEN-B database showed a slightly lower species-level annotation accuracy (50%) compared to GROND (58%). However, this direct comparison is not completely relevant. Indeed, the GROND sequences are longer than those of rEGEN-B, providing a better taxonomic resolution for CV. The GROND database also has a much wider taxonomic breath (e.g., it includes Archaea whereas rEGEN-B does not), which provides more context when querying the database and therefore increases the CV results. In addition, the sequences from GROND were clustered during the database construction (while rEGEN-B sequences were not), which may artificially enhance its accuracy.

**TABLE 3:** Databases included in PRONAME and comparison with other existing ones.

Database	Locus	Average sequence length (bp)	Number of sequences	Number of species	High-confidence curation*	References
Databases included in PRONAME						
Silva138	16S	1,457	436,680	48,933	No	Quast et al. (2012)
Greengenes2	16S	1,493	192,860	18,956	No	McDonald et al. (2023)
rEGEN-B	16S-ITS-23S	4,580	523,869	16,217	Yes	This work
rEGEN-B_uniq	16S-ITS-23S	4,614	115,032	16,217	Yes	This work
GROND	16S-ITS-23S	4,899	317,986	23,542	No	Walsh et al. (2024)
Databases from the literature						
RRN_DB2	16S-ITS-23S	4,073	493,329	11,762	No	Kinoshita et al. (2021)
MIrROR	16S-ITS-23S	4.260	97,781	9,485	No	Seol et al. (2022)
rOPDB	16S-ITS-23S	4,939	308,026	10,417	No	Kerkhof et al. (2022)

Two commonly used reference databases dedicated to the full 16S rRNA gene have been formatted and included in the PRONAME environment package (Silva138 and Greengenes2). For *rrn* operon sequences, a new curated reference database has been developed (rEGEN-B). For reduced computation times, an alternative database has also been built, by keeping only one *rrn* operon copy per genome (rEGEN-B_uniq). All these databases are available in the /opt/db/ directory. *: The “high-confidence curation” status reflects the fact that (i) the sequences in the database only come from genomes with confident assembly levels (i.e., “chromosome” or “complete genome” status, but not “contig” nor “scaffold”), (ii) only sufficiently recent genomes were retained for operon sequence extraction (nothing before 2005), and (iii) the database was curated using the DB4Q2 pipeline (Dubois et al., 2022) to discard low-quality and misidentified sequences.

### 3.2 Metabarcoding the mock community

**FIGURE 3:**
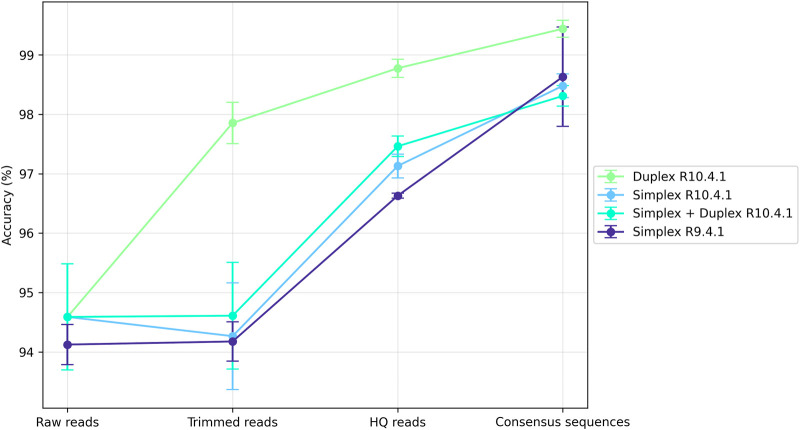
Read mean accuracy reached at different steps of the PRONAME workflow. Accuracy was computed for reads before their import into PRONAME (Raw reads), reads generated by proname_import (Trimmed reads) and proname_filter (HQ reads) and error-corrected sequences generated by proname_refine (Consensus sequences). The libraries were sequenced either with new V14 chemistry (R10.4.1 flowcells, producing both simplex and duplex reads) or with the older chemistry (R9.4.1 flowcells, generating only simplex reads). Each point in the graph represents the average of the values obtained in the four (R10.4.1) or the two (R9.4.1) relevant trials. The experimental design presented in Supplementary Material S3 was followed to produce this figure, using default parameters for every PRONAME script.

The metabarcoding sequencing data generated in this work were deposited on the NCBI under BioProject number PRJNA1139700. Details about the simplex/duplex read distribution among each sample, and length vs. quality plots can be found in Supplementary Material S4. Raw sequencing reads, as well as reads generated at different steps of the PRONAME pipeline, were blasted against the mock community genomes sequenced in this work. This enabled computation of the mean accuracy of these different read sets and illustrated the increase in accuracy across the whole pipeline (Figure 3). Overall, processing with PRONAME significantly increased read accuracy, regardless of the sequencing chemistry. With the current V14 chemistry (R10.4.1 flowcells), the results highlighted the benefits of the newly introduced duplex reads, which reached 99.5% ± 0.14% accuracy at the end of the PRONAME pipeline (with default settings, i.e. 90% clustering threshold). It was possible to attain even higher accuracy levels, like the 99% clustering threshold that led to an accuracy of 99.7% ± 0.03%. Detailed alignments of consensus sequences with their corresponding reference sequences extracted from the mock genomes are presented in Supplementary Material S5.

The results obtained with the older sequencing chemistry (R9.4.1 flowcells) were also interesting, given that similar accuracy to that of simplex V14 data (R10.4.1 flowcells) could be achieved. However, it was noticed that the quality of raw R9.4.1 data was poorer: the average read quality curve was centered around Q13 with almost no read above Q18, whereas V14 chemistry led to quality curves centered around Q15 and many reads between Q20 and Q30. Consequently, since the same quality/length parameters were applied for both R9.4.1 and R10.4.1 assays, this quality filtering resulted in much fewer HQ reads for R9.4.1 chemistry, which might not be ideal when aiming to detect low-frequency taxa.

### 3.3 The importance of the clustering step

**FIGURE 4:**
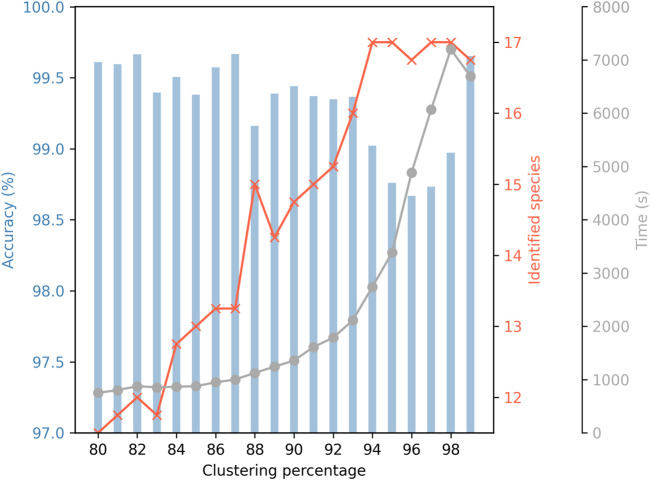
Influence of clustering identity threshold on accuracy, number of identified mock species and computation time. Accuracy refers to the mean identity percentage between consensus sequences generated by PRONAME and reference sequences extracted from the genomes of the species used in the mock community. Seventeen bacterial species were included in the mock community. Five of them belonged to the *Pseudomonas* genus and were deliberately chosen for their genetic proximity to put PRONAME to the test and evaluate its efficiency under very unfavorable conditions. The secondary grey curve represents the computation time needed to refine sequencing data and produce high-accuracy consensus sequences. To generate these results, raw data from the four sequencing assays that used V14 chemistry (RRN1 to RRN4 assays) were processed using PRONAME with increasing clustering thresholds from 80% to 99%. Default settings were used for the other parameters of the pipeline.

One of the key parameters in the PRONAME pipeline is the identity threshold selected to carry out the read clustering, as it affects the accuracy of consensus sequences, the number of identified species in the mock community, and computation time (Figure 4). The lower the threshold, the lower the number of generated clusters, and therefore the lower the number of centroid sequences to be processed during the time-consuming polishing step. The counterpart of using such low identity thresholds is that small variations may be overlooked, which explains the small number of identified species among the 17 species present in the mock community. It should be noted that, in cases where some species are missed, these are always *Pseudomonas*, and members of this genus are known to be difficult to differentiate genetically (Mulet et al., 2023). Conversely, using a high clustering identity threshold leads to more clusters, negatively affecting the computation time. Also, the clusters are smaller (with less reads used to polish the centroid sequence), which may explain the observed drop in accuracy (except for 99% where a significant increase in accuracy is observed). High clustering thresholds, however, allow identification of all species in the mock community, including the five *Pseudomonas*. Hence, these results illustrate how choosing the clustering identity threshold is a matter of compromise. This is the reason why the default value has been set at 0.90, as a middle ground between the number of identified species, computation time and accuracy achieved.

## 4 Discussion

### 4.1 Increasing nanopore metabarcoding accuracy and applicability

The PRONAME pipeline has shown to significantly increase the accuracy of Nanopore metabarcoding data thanks to several key characteristics such as read quality filtering, clustering and polishing, as well as the possibility of capitalizing on higher-quality duplex reads. With a duplex rate of at least 20% in our datasets, this provided more than enough duplex reads to cover the entire sequenced diversity. If, however, the user observes too few duplex reads in its data, working only with them may not be the most appropriate way to proceed, since the lower number of working reads could lead to an increased proportion of singletons after clustering. Given that singletons are removed at the end of clustering, this could represent a partial loss of useful information. If such a situation is faced, it is recommended to use both simplex and duplex reads. It must also be noted that our sequencing runs yielded on average 24,901±9,499 duplex reads and 229,214±22,164 simplex reads per assay. This number of duplex reads was sufficient to study the composition of the 17-species mock sample, but it may not be adequate for real-life samples, which probably contain many more species. In such cases, using both duplex and simplex reads might also be the most appropriate option. Admittedly, it will decrease the sequence accuracy of ∼1% (see Figure 3) but it represents ∼40–45 bp on a *rrn* operon amplicon (more than 4,000 bp), which should not significantly affect the outcome of the taxonomic assignments. The pipeline also enables valorization of older sequencing data by re-analyzing them, in light of the fact that processing reads from an older chemistry with PRONAME demonstrated a very satisfying increase in accuracy (Figure 3).

It is also important to understand how the clustering (and subsequent polishing) works to set relevant parameters according to the study context and research question. As highlighted in Figure 4, the clustering identity threshold had a significant impact on the average accuracy that could be reached, but also affected the number of species from the mock community that could be identified and, of course, the computation time. Although selecting a high threshold tended to decrease sequence accuracy, the extreme threshold of 99% showed a remarkable improvement and seemed to be a good option to obtain results combining good accuracy and fidelity in terms of identified species, provided the computation time is not an obstacle for the user. For more reasonable processing duration, thresholds of 90% (set as default parameter) or even 93% appear to be safe compromises. Indeed, both approaches achieved a high level of accuracy and identified a significant proportion of species in the mock community while maintaining computation time at a reasonable level. It is important to note that the mock community used in this work contained five *Pseudomonas* species, and it is known that members of the *Pseudomonas* genus are very difficult to differentiate genetically (Mulet et al., 2023). This explains why the number of identified species exhibits such a wide range according to the clustering threshold, from 12 to 17 (i.e., all) species in the mock community identified. It underlines the fact that this parameter should be set according to the study case, since this kind of complexity should not be expected for all types of samples. Similarly, this clustering threshold should also be adapted when working with different barcode regions, given that smaller clustering percentages could be selected if shorter amplicons were sequenced (Ohta et al., 2023).

### 4.2 Comparison to other existing pipelines

Different pipelines enabling the processing of Nanopore metabarcoding data have already been published. However, most of them do not incorporate an error-correction step with a Nanopore-dedicated tool. For instance, the MeTaPONT pipeline (Ammer-Herrmenau et al., 2021) basecalls fast5 into fastq files and then directly switches to taxonomic classification. The RESCUE pipeline (Petrone et al., 2023) includes additional steps like adapter/primer trimming and Q-score filtering but does not involve any error correction before the taxonomic analysis. The ASHURE pipeline (Baloğlu et al., 2021) does attempt to correct sequencing errors but not with a tool specifically developed for Nanopore data that takes into account its particular error profile. Instead, authors used an original approach of rolling-circle amplification (RCA) to generate consensus sequences that can reach median accuracies of up to 99.3% when long concatemeric reads can be achieved. However, the pipeline has been designed to process only such concatemeric data, which seems to be a niche application of Nanopore metabarcoding. It indeed requires an isothermal amplification, which is, according to the author’s words, a time-consuming step that may skew concatemeric reads toward shorter sequences. The Natrix2 pipeline (Deep et al., 2023) is very interesting as it includes, like PRONAME, clustering, chimera detection and error correction with Nanopore data-dedicated tools. Unfortunately, no information is provided about the accuracy that can be expected from consensus sequences generated by the pipeline. In addition, one major difference with PRONAME is the way sequences are polished to remove sequencing errors. Whereas PRONAME polishes a cluster centroid sequence with closely related reads (i.e., sub-reads from the same cluster), the whole initial sequencing reads are used for polishing in Natrix2. This approach was also evaluated in the present study but produced poorer performances with consensus sequence accuracy at around 92.5% (Supplementary Material S6).

The performance of PRONAME has also been directly compared to that of other pipelines. On the one hand, HTS raw data originating from assays RRN1 to RRN4 were processed using the Natrix2 and MeTaPONT pipelines to assess the sequence accuracy they could achieve. Compared to PRONAME, both pipelines displayed lower accuracy levels with 96.5% for Natrix2 and 94.7% for MeTaPONT (Supplementary Material S7). These levels can easily be explained by the reasons mentioned above, especially for MeTaPONT where the data processing is not as sophisticated as that of Natrix2 and PRONAME. On the other hand, sequencing data arising from ‘real-life’ samples were also analyzed using these three pipelines (Supplementary Material S8). The sequencing reads came from another study that investigated the composition of endophytic bacterial communities of tomato grown under sterile conditions in response to the application of osmotic stress and humic substances (Lengrand et al., 2024). The results provided by PRONAME highlighted the presence of *Bradyrhizobium*, *Sphingomonas*, *Methylobacterium* and *Ralstonia* species in control condition. When samples underwent osmotic stress and humic substance application, the appearance of *Frigoribacterium sp. SL97* was noted, combined with the disappearance of *Methylobacterium sp. FF17* and *Sphingomonas* species. These real-life samples were selected for their low bacterial diversity, confirmed by PRONAME, attributable to the sterile conditions in which tomato plants were grown. This low diversity is a strength here because it enabled verification that the tested pipelines do not produce false positives. Furthermore, the results provided by PRONAME align with those of previous studies, which showed identical species identification in other tomato cultivars (Wei et al., 2013; Shaik and Thomas, 2019). In contrast, both the MeTaPONT and Natrix2 pipelines were also able to successfully identify the major trends, but they produced additional less precise assignments, especially MeTaPONT, detecting a myriad of closely related species and unassigned sequences, reflecting remaining sequencing errors.

In addition, PRONAME displays other unique features. Firstly, in contrast to other pipelines, it does not require any installation or database download. Instead, it has been packaged in a Docker image that simply needs to be pulled from Docker Hub to run a container and proceed with sequence processing. All dependencies and up-to-date databases are precompiled in this Docker image. Secondly, PRONAME is the only pipeline aware of the new structure of V14 sequencing data, allowing taking advantage of duplex reads and their higher quality. Thirdly, whereas some pipelines do not perform clustering, this step is included in others but the way the assignment of reads to each cluster is done does not always seem clear or appropriate. In the Natrix2 pipeline for instance, raw reads are aligned against the consensus sequences to identify the read numbers per consensus, which represents more of an estimation than a precise count. In PRONAME, the exact number and sample origin of the reads constituting each cluster are recorded from the beginning of the clustering process. This generates an OTU-like table, gathering in one file the precise count of consensus sequences in every sample. In its structure, this table is identical to the OTU-/ASV-tables generated when using an Illumina metabarcoding approach, and therefore has the advantage of being useable for alpha-/beta-diversity metric calculation and differential abundance analysis among others. Lastly, the PRONAME pipeline has been designed to be used even without extensive bioinformatics skills. The commands are simple, documented, and the pipeline offers the possibility of importing the generated files into QIIME2, a user-friendly and probably the most widely used bioinformatics platform in the metabarcoding field.

## 5 Conclusion

The PRONAME pipeline has been developed to process Nanopore metabarcoding data and to significantly increase its accuracy and usability. Thanks to an innovative approach combining different quality filtering steps, read clustering, error-correction with a tool specifically dedicated to Nanopore data and the valorization of duplex reads, the results have demonstrated that the generated consensus sequences displayed at least 99.5% accuracy with default settings, and could reach 99.7%. The structure of the data produced by the pipeline allows direct advancement to further analyses such as inferring microbial diversity metrics between sample groups, or taxa differential abundance testing, for example,. Overall, this work represents a significant step forward in the field of DNA metabarcoding as the PRONAME pipeline very closely matches the accuracy of Illumina sequencing while taking advantage of Nanopore sequencing assets.

## Data Availability

The raw metabarcoding sequencing data generated in this work is available at the NCBI Sequence Read Archive under the Project Number PRJNA1139700. The sequenced genomes have been deposited on the NCBI under the BioProject Numbers PRJNA1134685 and PRJNA1141912. The PRONAME pipeline is available at https://github.com/benn888/PRONAME. The rEGEN-B database, already included in the Docker image, is also directly available at https://doi.org/10.6084/m9.figshare.26380702.
