# Supplementary figures and images for: PRONAME: a user-friendly pipeline to process long-read nanopore metabarcoding data by generating high-quality consensus sequences
Source: Front Bioinform. 2024 Dec 20;4:1483255. doi: 10.3389/fbinf.2024.1483255 (PMC11695402; doi:10.3389/fbinf.2024.1483255)

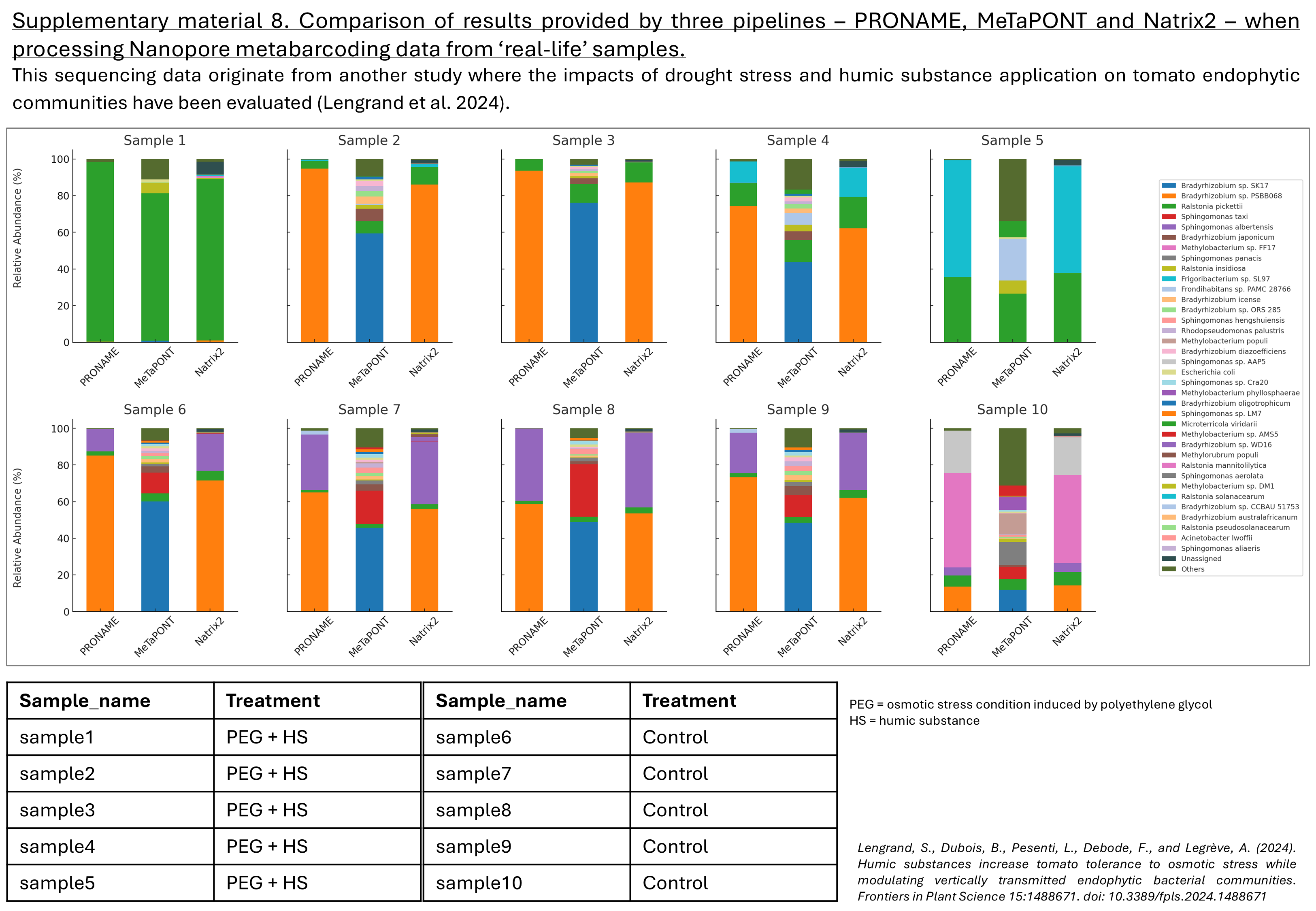

Supplement: Supplementary file 3 [file Image3.tif]

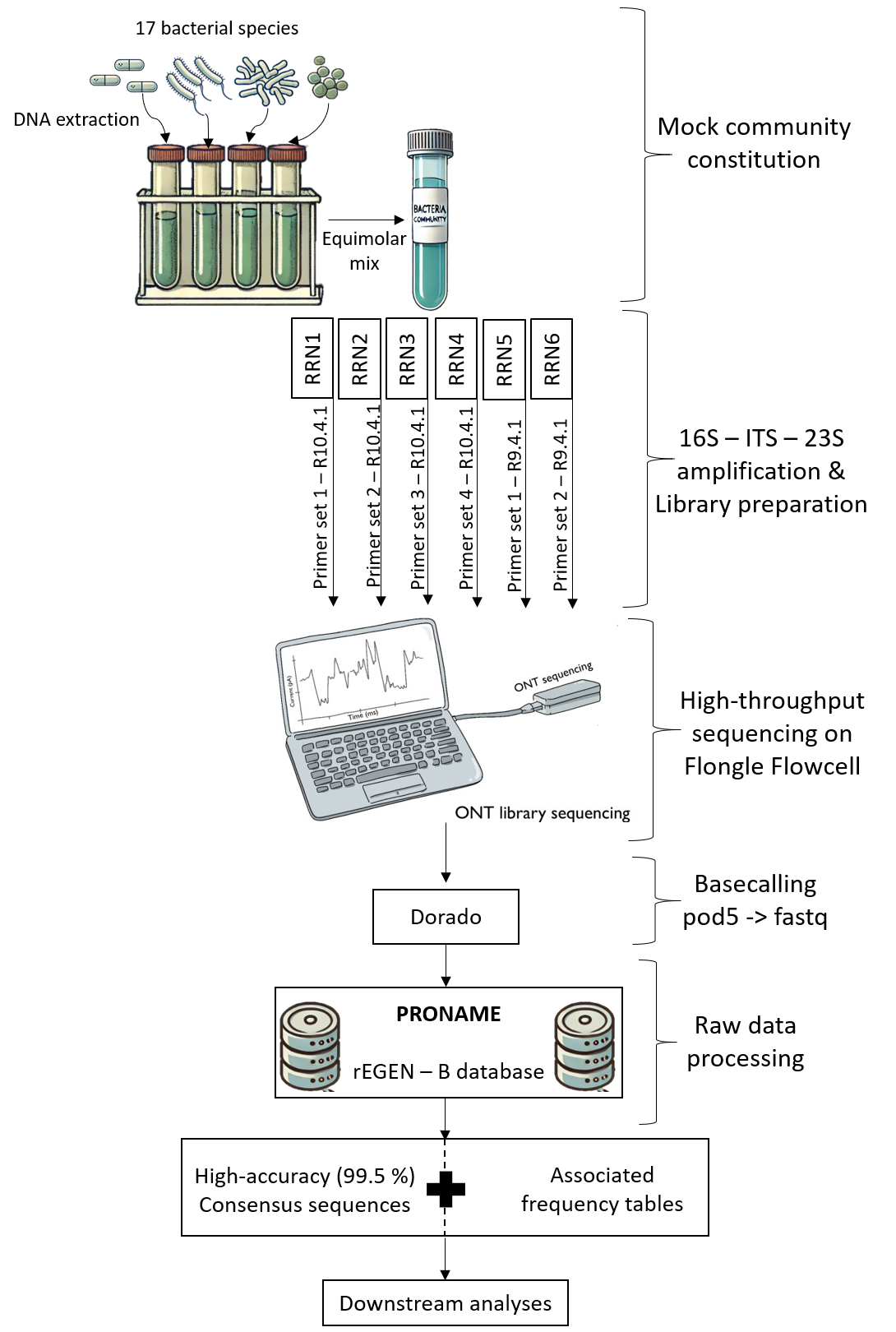

Supplement: Supplementary file 4 [file Image1.tif]

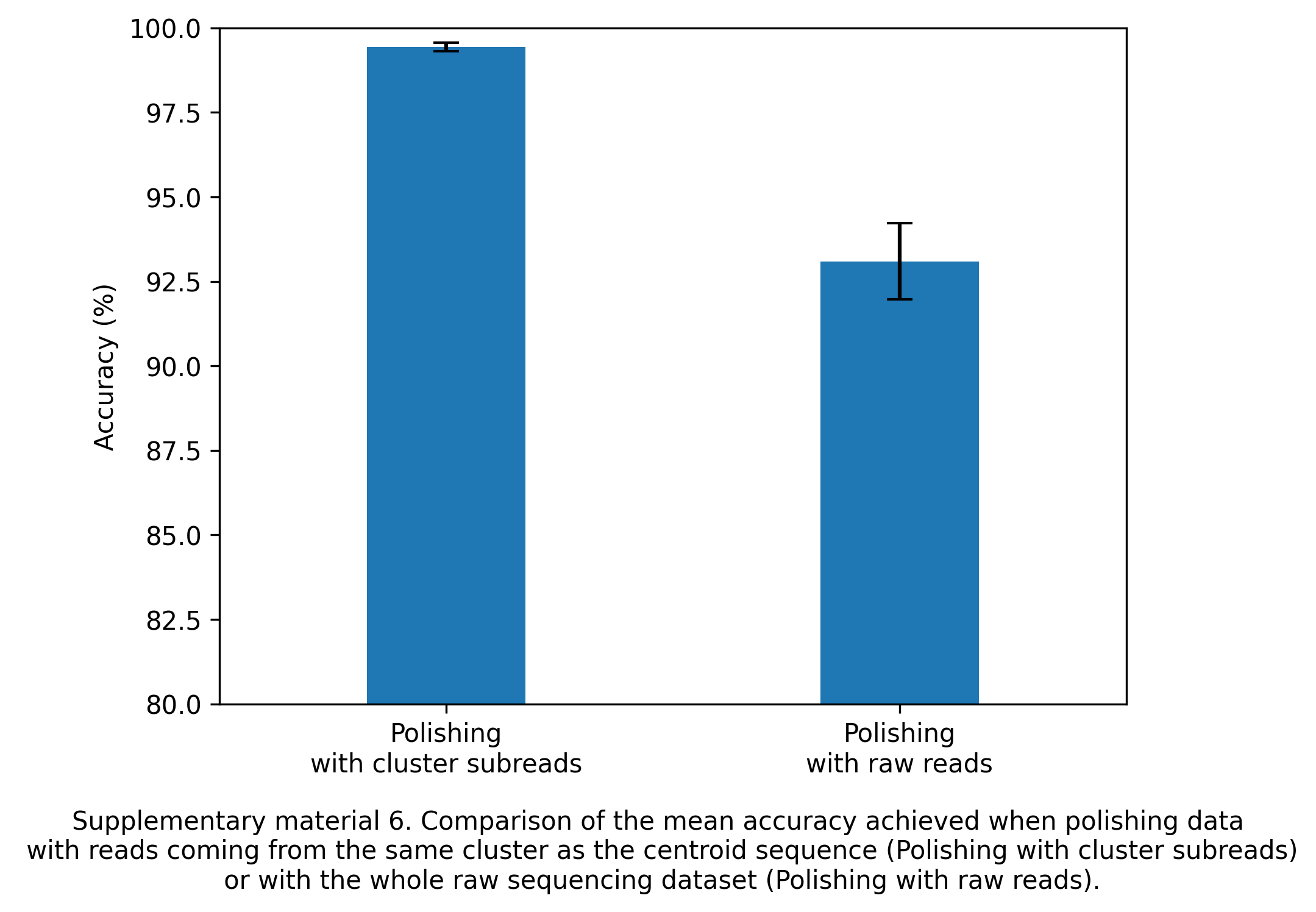

Supplement: Supplementary file 8 [file Image2.tiff]
