# Supplementary material for: PRONAME: a user-friendly pipeline to process long-read nanopore metabarcoding data by generating high-quality consensus sequences
Source: Front Bioinform. 2024 Dec 20;4:1483255. doi: 10.3389/fbinf.2024.1483255 (PMC11695402; doi:10.3389/fbinf.2024.1483255)
Supplement: Supplementary file 5 [file DataSheet1.pdf]

Collection_number	Kingdom	Phylum	Class	Order	Family	Genus	species	Gram
UPB 1356	Bacteria	Bacillota	Bacilli	Bacillales	Bacillaceae	Bacillus	subtilis	+
LMG 22950	Bacteria	Pseudomonadota	Betaproteobacteria	Burkholderiales	Burkholderiaceae	Burkholderia	anthina	-
UPB 1352	Bacteria	Pseudomonadota	Gammaproteobacteria	Enterobacterales	Enterobacteriaceae	Enterobacter	kobei	-
UPB 1350	Bacteria	Actinomycetota	Actinomycetes	Micrococcales	Micrococcaceae	Glutamicibacter	creatinolyticus	+
LMG 24788	Bacteria	Pseudomonadota	Alphaproteobacteria	Hyphomicrobiales	Methylobacteriaceae	Methylobacterium	bullatum	-
UPB 460	Bacteria	Actinomycetota	Actinomycetes	Micrococcales	Microbacteriaceae	Microbacterium	oxydans	+
UPB 1357	Bacteria	Pseudomonadota	Gammaproteobacteria	Enterobacterales	Erwiniaceae	Pantoea	agglomerans	-
LMG 31463	Bacteria	Bacteroidota	Sphingobacteriia	Sphingobacteriales	Sphingobactericeae	Pedobacter	foliorum	-
UPB 461	Bacteria	Pseudomonadota	Gammaproteobacteria	Pseudomonadales	Pseudomonadaceae	Pseudomonas	cichorii	-
UPB 526	Bacteria	Pseudomonadota	Gammaproteobacteria	Pseudomonadales	Pseudomonadaceae	Pseudomonas	asplenii	-
UPB 1355	Bacteria	Pseudomonadota	Gammaproteobacteria	Pseudomonadales	Pseudomonadaceae	Pseudomonas	lurida	-
UPB 1354	Bacteria	Pseudomonadota	Gammaproteobacteria	Pseudomonadales	Pseudomonadaceae	Pseudomonas	sivasensis	-
UPB 463	Bacteria	Pseudomonadota	Gammaproteobacteria	Pseudomonadales	Pseudomonadaceae	Pseudomonas	syringae	-
UPB 1353	Bacteria	Bacteroidota	Sphingobacteriia	Sphingobacteriales	Sphingobactericeae	Sphingobacterium	thalpophilum	-
LMG 32139	Bacteria	Pseudomonadota	Alphaproteobacteria	Sphingomonadales	Sphingomonadaceae	Sphingomonas	albertensis	-
UPB 1351	Bacteria	Bacillota	Bacilli	Bacillales	Staphylococcaceae	Staphylococcus	equorum	+
UPB 513	Bacteria	Pseudomonadota	Gammaproteobacteria	Xanthomonadales	Xanthomonadaceae	Xanthomonas	translucens	-

Supplementary material 2. List of the 17 bacterial species used to develop the mock community.
Five of them belonged to the *Pseudomonas* genus and were deliberately chosen for their genetic proximity to put PRONAME to the test and evaluate its efficiency under very unfavorable conditions.
